# A Study of the Performance Degradation of Conductive Threads Based on the Effects of Tensile Forces and Repeated Washing

**DOI:** 10.3390/polym14214581

**Published:** 2022-10-28

**Authors:** Adrian K. Stavrakis, Mitar Simić, Goran M. Stojanović

**Affiliations:** Faculty of Technical Sciences, University of Novi Sad, Trg Dositeja Obradovića 6, 21000 Novi Sad, Serbia

**Keywords:** conductive threads, tension testing, washability, textile electronics, SEM imaging, flexible electronics, textile electronics

## Abstract

In recent years, after the ongoing success in the creation of portable electronic devices, an increasing effort has been put in creating wearable devices capable of sensing multiple parameters while being imperceptible to the user. A field that has recently gained attention due to this is that of textile electronics. For this purpose, one of the most commonly used materials is conductive threads, capable of sustaining an electrical connection, while at the same time being part of a garment. As research on the performance and stability of such threads is scarce, the aim of this work is to study the effects of tension on readily available conductive threads and to verify their suitability and reliability for e-textile applications. After testing seven commercially available threads, this study demonstrates that the nominal parameters provided by the manufacturers are not in line with experimentation, and that both embroidery and washing have an impact on their performance.

## 1. Introduction

Smart wearable devices have attracted enormous interest in recent years. Aside from commonly known commercial products such as smartwatches, research has been ongoing in enabling comfortable real-time sensing of various parameters. Even though new fabrication techniques have allowed for flexible devices, most materials used are still not compatible with on-body utilization, or are still uncomfortable to the user, with the exception of conductive textiles and threads.

In terms of new fabrication methods, the utilization of flexible substrates is prevalent, allowing for wearable devices to conform to the curvature of the body. Naturally, the well-established processes of subtractive and additive manufacturing utilized for conventional electronics (such as wafer coating, etching, chemical vapor deposition, etc.) cannot apply to materials such as textiles both due to their surface properties and limitations. The assembly of all the required electronics on these devices is performed using stamp transferring [1], photolithography [2], ink printing [3,4], microdispensing [5], and most prominently screen printing [6,7,8,9]. Even though regular polymer thin films are often used, their breathability is limited owing to their non-porous nature, so alternatives such as fabrics are being currently researched. Common methods of supplying power are triboelectric generators [10], body-heat harvesters, thermoelectric generators [11], wearable solar cells [12], and electrochemical cells [13,14].

Lastly, in terms of new materials, the task of forming electrical interconnects is primarily vested upon conductive pastes and conductive threads, with the latter being significantly easier to work with as they are not impacted by backing fabric morphology. In addition, when compared to other conductive-fabric fabrication technologies, such as plating a textile with a conductor [15], they are significantly cheaper and much faster to work with, as no specific screen, expensive equipment, or multistage fabrication process is required.

This is why there are a multitude of applications and research work already that relies on them, primarily in the field of sensing, with examples such as zinc oxide nanowire-decorated threads [16], stretchable gold-fiber-based wearable sweat nano-biosensors [17], perspiration level monitoring sensors [18], and textile multimodal sensing devices [19]. Another field of conductive-thread usage is heating, with a coil based on conductive threads for an induction wearable heater being developed in [20], and a wireless heating textile for thermotherapy in [21].

In addition to this, other precision applications of conductive threads include embroidered antennas [22,23] and an investigation on the challenges of such fabrication [15], a wireless power transfer system reliant on conductive threads [24], and chipless RFID tags [25]. As RFID tags and antennas have very precise fabrication requirements in terms of conductor resistance and stability, many phenomena can occur when this changes, such as drops in efficiency, resonating frequency slips, beamwidth alterations, etc., as observed in [26] after repeated washing of a bow-tie ISM-band antenna.

It is, therefore, evident, that with the rise in the popularity of conductive threads as a material for embroidered electrical and electronic applications, such threads should be studied in terms of their mechanical and electrical performance and stability, so they are better placed for other potential applications close to their limitations. Moreover, due to the multitude of techniques for the fabrication of conductive threads, and the plethora of materials used [27], it is crucial to establish the material limits; however, current scientific research has not yet addressed these issues, which is the scope of our paper.

In our manuscript, seven different commercially available conductive threads were studied to verify their mechanical properties under a tension testing system, as well as under the effects of embroidery and washing. Simultaneously, they were tested in terms of their electrical performance by measuring their resistance per meter to verify their behavior and stability after elongation, embroidery, and washing. A statistical analysis was also carried out on the sample population, and the key observations obtained were the appearance of delamination and microfracturing of the silver coating; a general increase in the resistance of threads after tension and washing; and a slight deviation from the manufacturer’s provided values for the maximum force at break, elongation at break, and resistance per meter across all threads.

## 2. Materials and Methods

### 2.1. Studied Conductive Threads and Common Testing Methods

Currently, there are only a few readily available conductive-thread products, as their manufacturing progress is significantly more complicated than that of regular sewing threads. Luckily, two of the major thread manufacturers have already launched their own lines of conductive threads; these manufacturers are Madeira and Amann Group. For this study, the entire conductive-thread catalog of both manufacturers was selected to be put under test.

The product line of Madeira consisted of only two threads, composed of silver-coated polyamide, while the catalog of Amann Group contained two series, one composed of silver-coated polyamide (2 products) and one of a silver-coated polyamide/polyester hybrid (3 products). Table 1 summarizes the key properties of all studied threads, as found in the datasheets of each manufacturer [28,29,30].

In general, standardization targeting specifically the field of technical textiles, textile electronics, textronics, or that described by any other utilized nomenclature is currently either under discussion or totally absent. Therefore, the manufacturers rely on existing DIN EN ISO standards to provide values for the linear density (2060:1994) of their threads, the maximum force at break, and the percentage of elongation at break (2062:2009).

It is worth noting that for both manufacturers, the values for the breaking force and percentage of elongation at break were calculated using the DIN EN ISO 2062 standard. However, as in a single cone of thread one can find a few kilometers of length, it was crucial to verify whether these calculated values adhered to or were subject to production instabilities. In general, DIN stands for the national standard, EN stands for the European standard, and ISO serves as the international standard. However, in the case of the ISO 2062 with its latest active revision from 2009, which replaced the previously active version of 1993, all the DIN, EN, and ISO variants have been unified in Europe. As per the scope of this work the determination of the linear density was not important, no research has been carried out on whether the 1994 version of the 2060 ISO standard is also in unison with the DIN and EU variants [31].

### 2.2. Tensile Testing in a Lab Environment

Given the above, to verify the accuracy of the values presented in the manufacturer datasheets with respect to the maximum force at break and elongation percentage at break, a tension testing system (34SC-2, INSTRON, Norwood, MA, USA) capable of replicating the standard was utilized to evaluate 50 individual samples of each thread. This model had a 2 kN force transducer, which was well above the minimum force margin requirements from the standard. These samples were of a 250 mm length between the grips of the instrument, and were tested at a constant rate of extension of 100% per minute, i.e., 250 mm/min.

In line with the ISO 2062:2009 standard, to ensure no slippage of the samples during testing, but at the same time to avoid their breakage at the gripping points, the standard set of flat grips was changed to a set of rubber-coated flat grips. After collecting all data on all fifty samples per thread, a linear regression (fitting) was performed on each thread’s data, at the minimum, maximum, and average values recorded. In addition to this, goodness of fit data, namely, the *R^2^* and *Sy.x* values, were extracted as:

(1) $$R^{2}=1-\frac{SS_{residual}}{SS_{total}}$$

(2) $$Sy.x=\sqrt{\frac{\sum {\left(residual\right)}^{2}}{n-K}}$$

with *SS* being the sum of squares, *n − K* being the number of degrees of freedom of the regression, and *K* being the parameter fit with the regression.

Following this, a new sample volume of twenty samples per thread was used to determine the maximum percentage of elongation up to which the threads could retract to their original length. The same sample volume was used to measure the resistance-per-unit-of-length change per thread, and for this purpose, one measurement was conducted on each sample before testing, and a second measurement after the sample reverted to its original length after tension application.

To perform this second test, since the experimental breaking points per thread were already established, the new samples were loaded into the tension testing system one by one, and each time, a force was applied to them in increments of 5% of the total average breaking force. Then, the force was released, and the instrument was brought back to zero displacement. If the sample at the 0-displacement position showed signs of relaxation, it was considered that the deformation was permanent, alternatively, the test was repeated with an extra 5% of the total breaking force, until all samples reached permanent deformation.

### 2.3. Testing in “Real-Life” Conditions

An important aspect of wearable electronics, particularly of the e-garment type, is their ability to withstand being washed. There are two main potential degrading factors during a washing cycle. Primarily, the various forces exerted on the garments during the wash cycle, either centrifugal, frictional, or impacts, and secondly, to a lesser extent, the effects of the chemicals and surfactants found in the washing supplements such as powders, liquids, softeners, etc. Towards this end, a washability test was designed to evaluate the performance of such threads. As a free-standing thread would be washed out of the drain of a commercial washing machine due to pump and centrifugal effects, a minimally invasive way of attaching the conductive threads to a piece of fabric was crucial.

**Figure 1:**
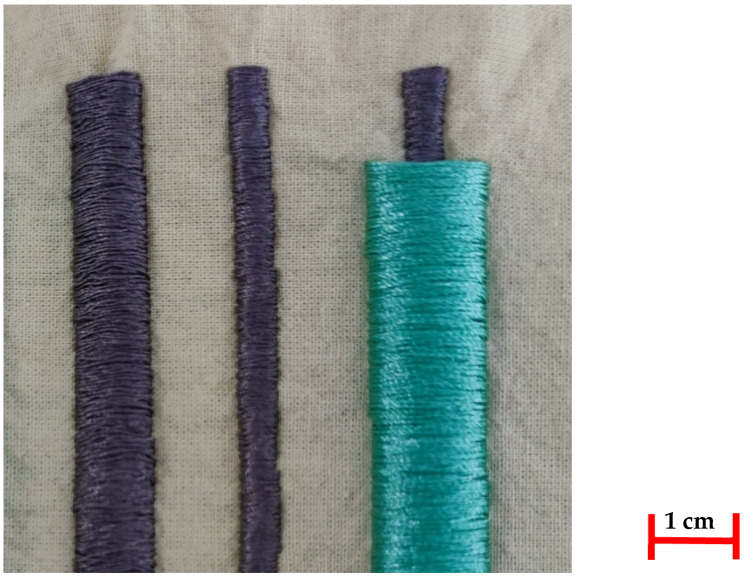
The embroidered conductive-thread patches in the two variations, with the control pattern on the left-hand side.

To achieve this, and by considering the length necessary to replicate the experiment from Section 2.2, rectangular patches were created in a technical embroidery machine (JCZA 0109-550, ZSK, Krefeld, Germany). The peculiarity of these patches was that instead of a normal satin or complex stitch, which would create multiple attachment points with the backing fabric and the bottom thread, jump stitches were implemented, so that the thread would only be attached at the two parallel ends of each rectangle. The patches were created in two variations. One covered with a polyamide thread, and one without (Figure 1). These patches were produced in eleven replicas, out of which, one was used to only determine the effect of embroidery tension on the performance, and the rest of the eleven, to study the effects after one to ten cycles, respectively.

The wash cycles were carried out using a household washing machine, in a mixed garment 2 kg load, at a 30 °C temperature, and a program of 30 min duration. The pieces of fabric containing the thread patches were placed inside a separate washing machine bag. The spinning cycle speed of the selected wash cycle was 600 rpm. The hardness of Novi Sad water was approximately 15 dH (German degrees of hardness) and as such it was characterized as medium hard. The fabrics were then dried naturally on a clothes airer. Following this, the stitches were broken so that the full length of the thread constituting the patch was released from the garment, and then utilized in the tension testing machine using the method described in Section 2.2. Samples were also extracted to be studied under scanning electron microscopy.

## 3. Results

### 3.1. Performance of Individual Threads under Tension

**Figure 2:**
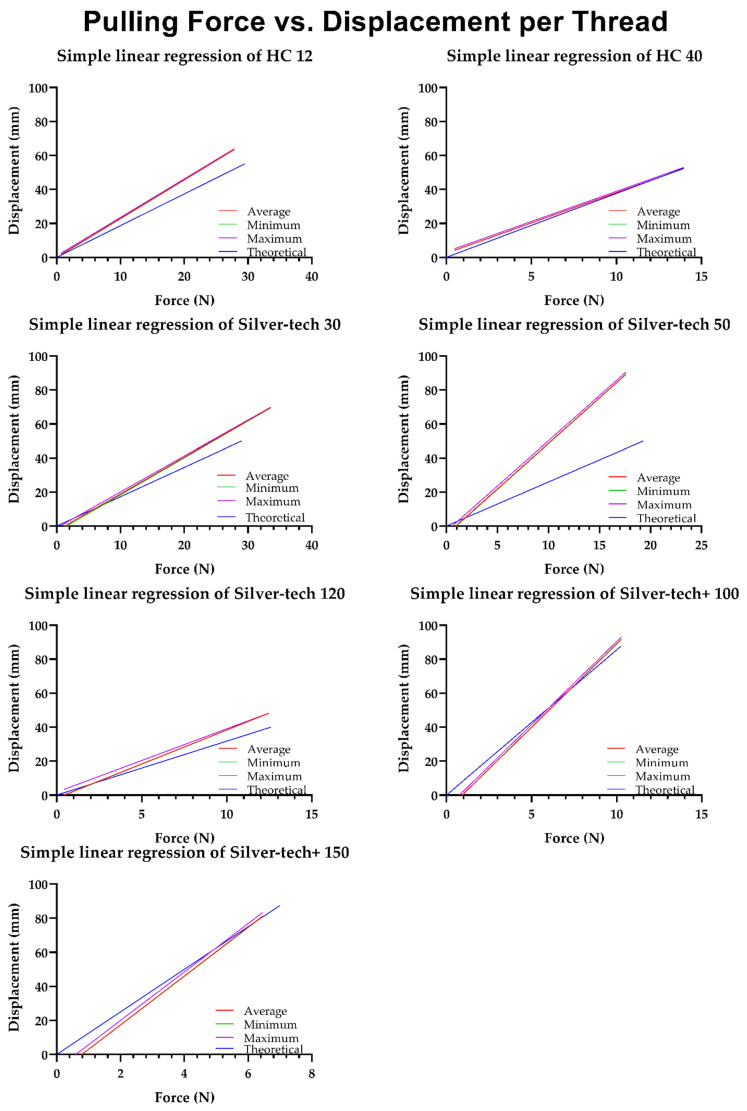
Linear regressions and theoretical line plots of the force–displacement relationship per thread type.

As described in the method section above, to meet the ISO 2062:2009 standard requirements, fifty samples of each thread were subject to tension testing until fracture. Figure 2 presents four lines per thread, one fitted at the maximum values recorded, one at the minimum, and one at the average. The fourth line links graphically the point (0,0) to a point (X, Y) with X, Y representing the force at break provided in the datasheet and the percentage of elongation at break converted to the displacement of a 250 mm sample, respectively.

The process of converting the value of the percentage of elongation at break to the displacement was a simple unit reduction process where, if a sample had a length of 100 mm, a y% elongation at break meant that its new length was 100 mm + y mm and its overall displacement was y mm. As the samples for this work were all at a length of 250 mm, the same y% elongation was converted to a displacement (mm) as follows:

(3) $$Displacement\;\left(\mathrm{mm}\right)=\frac{y\%\;\left(\mathrm{mm}\right)\times 250\;\mathrm{mm}}{100\;\mathrm{mm}}$$

**Figure 3:**
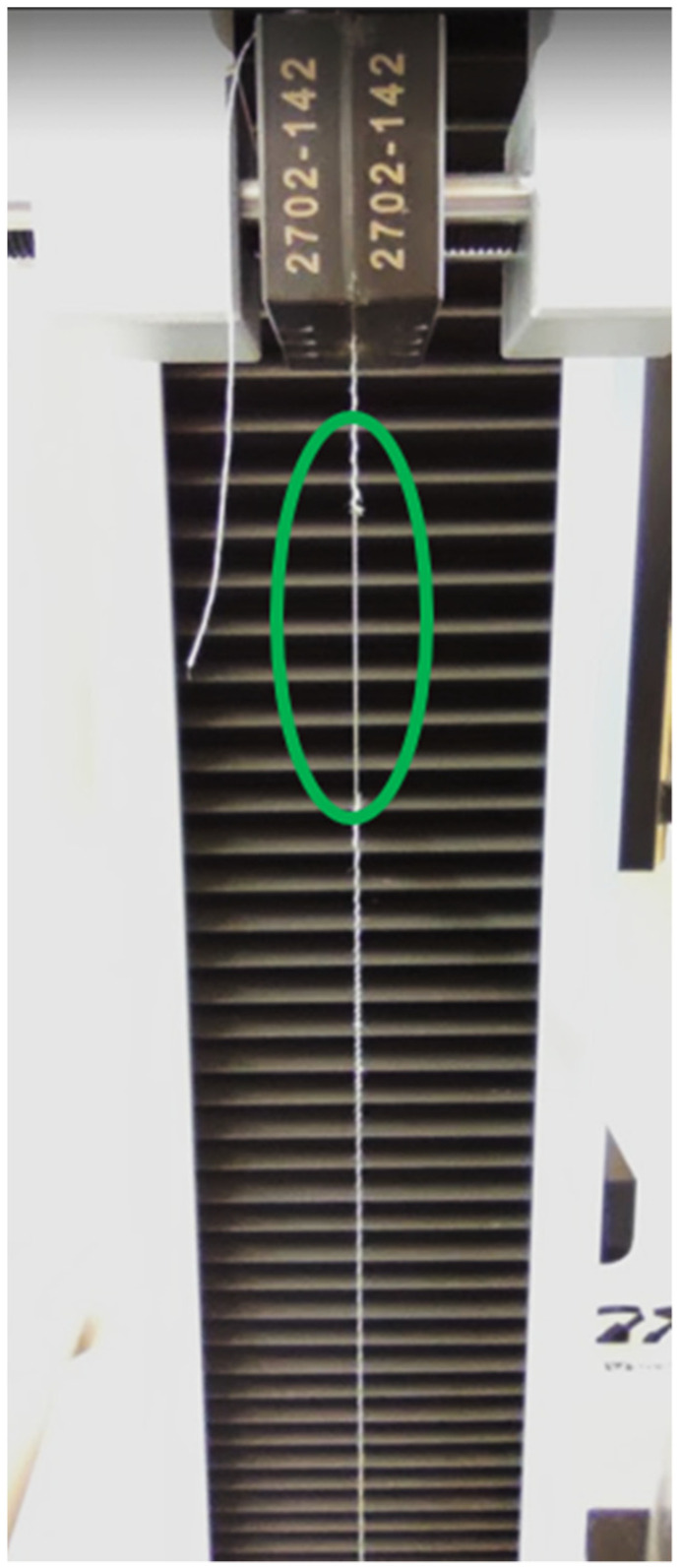
A sample of Silver-tech 50 tensioned beyond its first-stage fracture, still partially intact. The affected area is enclosed in the oval.

It was immediately evident that the fitting lines of the minimum and average values overlapped almost entirely among all threads, with a slight exception in the case of the Silver-tech 30. This denoted that, from the total of fifty samples per thread type, most of them were producing series of points closer to the minimum line than the maximum, hence, the average was shifted towards it. From Figure 2, it can also be seen that in the case of the Silver-tech 50, all three fitted lines were the farthest away from the theoretical one, and this was in conjunction with the table data (Table 2), which showed a significantly worse R^2^ value of approximately 0.45 versus the minimum of 0.88 for all the other threads. This was attributed to a strange behavior present only on this thread under testing, under which the thread would snap, but not entirely, and then after continuing tension, it would snap again, this time entirely (Figure 3).

It is also worth mentioning that among the tested models, the most stable thread seemed to be the Madeira HC 40, with all three fitted lines almost perfectly overlapping while also being the closest to the theoretical one, and with the closest slope exhibited among all threads. This was also verified with the goodness of fit data, as it displayed the highest R^2^ values among all threads, and the lowest Sy.x. This performance was also followed closely by the other Madeira thread, HC 12, even though it was not able to tolerate—on average—the maximum theoretical force at break.

**Figure 4:**
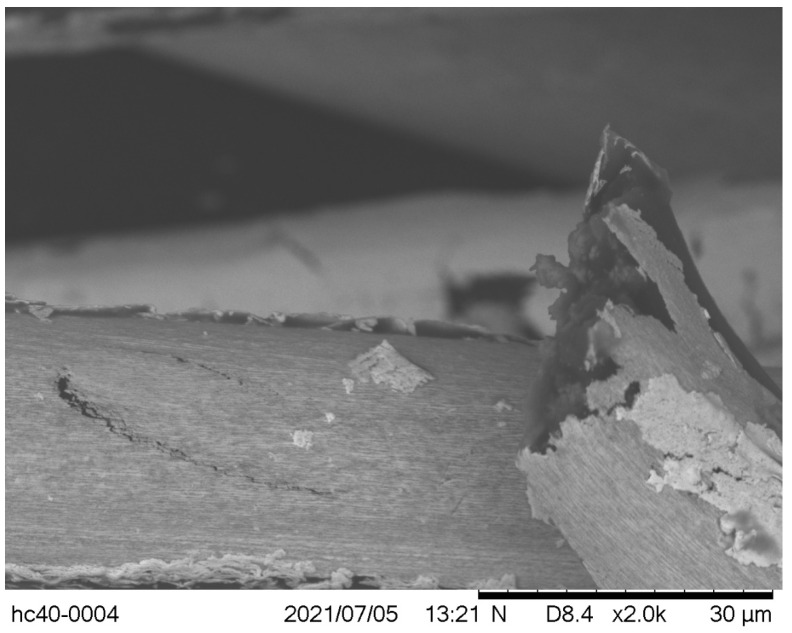
A sample of HC 40 post-fracture. The silver coating is severely damaged but not entirely released from the polymer core, except at breaking point.

In terms of damages sustained, all fractured threads were studied under scanning electron microscopy (SEM) (Hitachi TM3030, Tokyo, Japan), to visualize any key details post-fracture. One key outtake was the fact that the silver coating was dislodged at the point of fracture, however, it had not entirely disappeared from the polymer surface. This can be seen in Figure 4, where at a magnification scale of ×2000, on the right-hand side, a fractured fiber of the Madeira HC 40 is shown. Its core, dark grey in color, was exposed at the point of fracture, but except for the bottom-right corner where a piece of the original coating remained, all the remaining fiber was coated with a very thin layer of silver, hence, it did not present in the same dark-grey color. Simultaneously, the unsevered fiber, which runs horizontally at the bottom of the image, had also lost most of its silver coating, while at the top part of it, one can observe the remaining of the silver-coating layer thickness ready to peel off.

**Figure 5:**
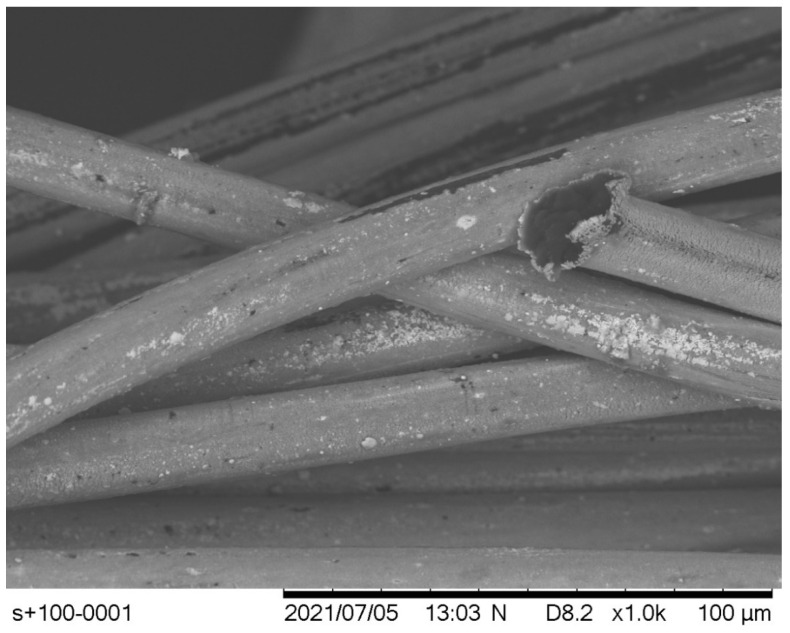
A sample of Silver-tech+ 100 post-fracture. Once again, the severely damaged silver-coating layer has not entirely disappeared after the break.

This was an omnipresent behavior among all the fibers and threads, as comparable results can be seen in Figure 5, showing a sample of the Silver-tech+ 100 series once again having shed most of its silver coating, except for some parts directly below the severed fiber end pictured. In the same figure, another observation could be made, but this did not apply to the two threads produced by Madeira as they did not demonstrate such behavior. It was observed that at the point of fracture, the cross-sectional area of the thread had expanded permanently, with the break resembling a mushroom-like shape. This was in line with the findings of Rengasamy and Wesley [32] and the attribution of this occurrence to the generation of heat due to the localized transverse stresses, high-speed breaks, and/or the exposure of certain filaments to the elongation force suddenly, following the breakage of other filaments prior to this.

Lastly, by comparing the axis intercept point at the end of the average fitting line with that of the theoretical line and converting back the displacement to the percentage of elongation at break, Table 3 provides a clear picture of the performance of each thread.

### 3.2. Effects of Tension on the Permanent Deformation of the Threads

After having established the limits of each thread, twenty new samples of each were used to determine at what point of applied tension the elongation became permanent. For this purpose, the sample was loaded into the tension testing system and was pre-tensioned to a value of 1 N. Then, the instrument was instructed to displace the sample until 5% of the average breaking force for the tested thread was reached and then reverse to the original position. If the sample was still tensioned (causing a value > 0.9 N at the origin), the process was repeated to a displacement of 10% and reversed, until the sample no longer satisfied the pre-tension condition, signifying that it was irreversibly elongated. The results of this study can be seen in Table 4.

**Figure 6:**
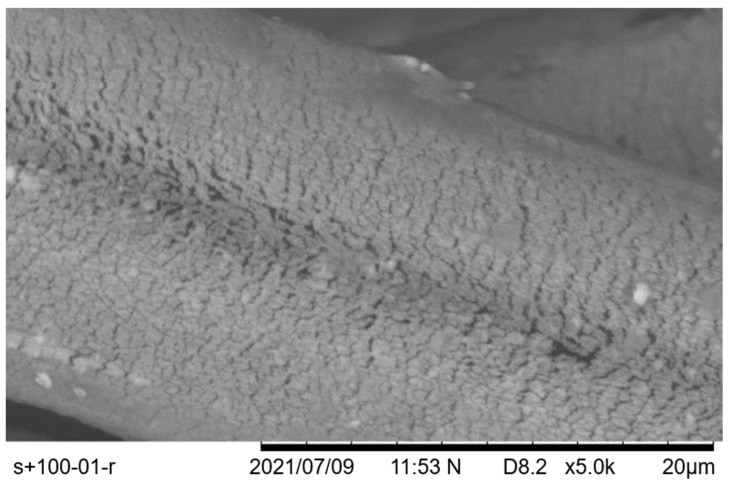
SEM imaging of Silver-tech+ 100 threads after tensioning to the level of permanent elongation, with visible microfractures of the silver coating.

As expected, threads of higher density were generally stiffer and sustained more tension before suddenly breaking, without any significant permanent deformation. However, after observing the samples under scanning electron microscopy, as can be seen in Figure 6, even though they were possibly not broken, the silver-coating layer exhibited significant microfractures along the entirety of its surface. It should be noted that this time the effect was different, as these fractures extended all the way to the core of the thread, in contrast to previous damages from breaking which retained a thin uniform part of the silver coating attached to the polymer. This was a very important finding, as in the first case, in terms of electrical conductivity, it was expected that the nominal resistance value of the thread would rise due to the thinner conductive silver layer; however, in the second case, the problem became very complex, as these microfractures voided the assumption that the material was uniform by being active electrical discontinuities. In the supplementary material section, further Scanning Electron Microscopy images can be found, detailing each thread under two different magnifications.

### 3.3. Impact of Tension in the Resistivity of the Threads

Each of the samples per thread were subject to a resistance measure before testing and after the point where the deformation was permanent. As previously, the samples tested were 25 cm long, and the testing was carried out using a digital multimeter (PC510a, SANWA, Tokyo, Japan) after they were cut to size. Then, the value was converted to the corresponding per-meter value in order to be easily compared with the datasheet. As the values for resistances in the datasheets were provided for all threads using meters as a unit of length, Table 5 shows the minimum, maximum, and average resistance per thread before and after tension was applied. A resistance measurement was not taken in the case of verifying the force and elongation at break values in the first step, as the samples were pushed to catastrophic failure to produce the required values.

In conjunction to the findings about the silver-coating microfractures (Figure 6), an expected rise in the resistivity value was validated experimentally. However, due to the existent discontinuities, it could not be assumed that it was a uniform change in the thread (a spool of which came in the range of km in length). Because of this, an extensive investigation should be carried out should one want to transmit AC signals through such threads, as in this domain, microfractures can give rise to a multitude of adverse effects.

### 3.4. Impact of Washing on the Resistivity and Elasticity of the Threads

A first assessment of the impact of washing on the performance of the studied conductive threads was that of comparing the mean force at break of healthy samples to that of embroidered samples, and to that of samples washed from one to ten times. The number of ten washes was chosen as a maximum primarily to save on resources such as water and energy, but at the same time, it allowed any performance degradation of threads to manifest.

**Figure 7:**
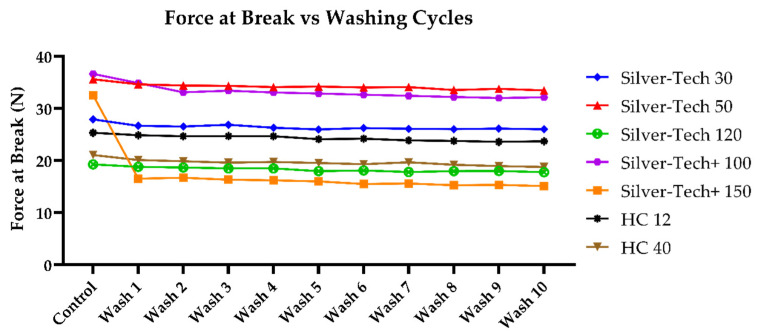
Change in force at break after washing across all tested thread samples without protective polyamide thread on top.

As can be seen in Figure 7, all threads could tolerate a slightly less maximum force at break. This could be attributed to the effects of washing which may have acted as an abrasive, not only in terms of constant tumbling and impacting, but also due to the force of the washing water hitting the threads repeatedly. A notable difference that could further sustain the previous assumption was observed in the case of the Silver-tech+ 150, the force tolerance of which dropped significantly more than the rest of the population, likely attributed to its smaller diameter, and by extension fewer individual fibers spun together. An abrasion could theoretically be measured under SEM; however, the thread samples were not only tubular, but also co-spun, which limited the ability to precisely measure the diameter of each.

**Figure 8:**
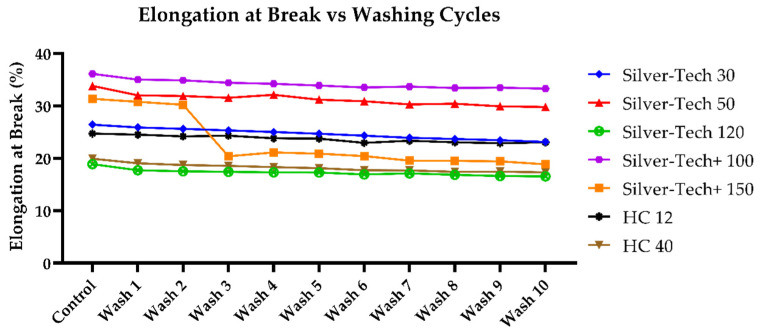
Change in elongation at break after washing across all tested thread samples without protective polyamide thread on top.

Additionally, the same comparison was performed for the mean elongation at break, as seen in Figure 8. Once again, the maximum elongation at break was slightly decreased. A curious behavior, however, was that of the Silver-tech+ 150, which abolished its characteristic partial break and was breaking in one step, as the rest of the fiber population.

It was found that for the entirety of the thread population, the maximum sustained elongation gradually decreased with the increasing number of washes. Even though this change was within 15%, which could be considered within the acceptable tolerance for many applications, it was still worth considering that this effect was discovered within only ten washing cycles and was present since the first iteration. Assuming a combination with actual thread utilization in a real-life application, which would subject it to further stresses, a further performance degradation should be expected.

**Figure 9:**
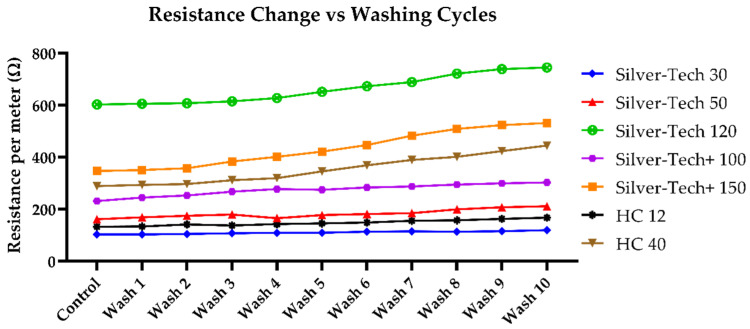
Change in average resistance-per-meter value after washing across all tested thread samples without protective polyamide thread on top.

Lastly, the same study was conducted in terms of the electrical resistance changes (Figure 9). A similar resistance change was observed in the case of tension effects, i.e., an increase in the resistance values per meter across all threads. Moreover, it was observed that the thinner threads tended to have a more prominent resistance change. This could be attributed to the fact that less individual fibers were present in these threads, which lead to a larger part of the total being exposed to the wash effects, rather than being on the inside of the co-spun batch that formed the thread. The difference between the measured values of the resistance changes between the batch that was covered with a protective polyamide thread layer and the uncovered one was negligible. However, under SEM analysis, no significant structural differences were observed versus the tensioned but unwashed samples.

**Figure 10:**
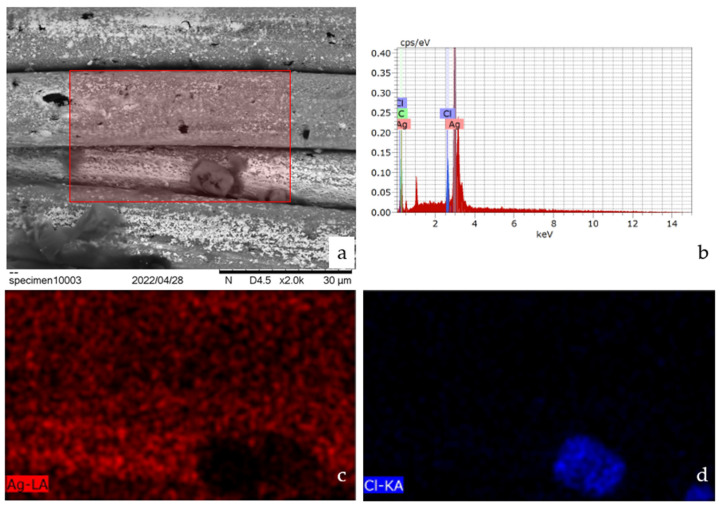
SEM image of the washed thread with the EDX margins highlighted (**a**), EDX spectra (**b**), silver (**c**), and chlorine (**d**) mapping at a ×2000 magnification.

Furthermore, an EDX analysis showed that there were only trace amounts of elements found in surfactants or water treatment (Figure 10). Therefore, in absence of attached particulates on the fibers after washing, either with visual confirmation or EDX, it could be inferred that the change in the thread stiffness could only be attributed to the effects of the forces during the washing cycle. A series of tumbles and impacts might have gradually changed the winding of the fibers on the free sides, inducing internal stresses on the individual fibers, or potentially centrifugal forces might have caused the threads to behave like a catenary, being “pulled” at the center which was free due to the utilization of jump stitches, causing fatigue towards the fixture points, which may have impacted the force carrying capacity of the thread. Further investigation of the matter was proposed, however, this required specialized techniques, such as X-ray tomography, in order to assess the fiber positioning and tension while it was still attached to the backing fabric, and not after it was released.

## 4. Discussion

This work examined seven different conductive threads in terms of their performance stability and degradation under mechanical forces induced both with a specialized tension testing system, as well as from repeated washing cycles. It was interesting to see that across all threads, the values for the elongation at break provided by the manufacturers tended to underestimate the real thread tolerance to breakage, varying from 15.6% in the case of the Silver-tech 50, which curiously failed in two stages, to 0.2% in the case of the HC 40. In addition to this, the force at break was overestimated for the majority of threads, although the differences were confined within ±2 N, with the exception of the Silver-tech 30, which was able to tolerate forces up to 33.9 N instead of the 29 N advertised.

The effects of degradation due to repeated washing were similar, with an interesting finding being the almost total absence of foreign material adhered to the threads, such as residue from detergents, dirt trapped in the insides of a washing machine, etc. This could be potentially attributed to the metalized coating of the studied threads, which also did not seem to be extremely negatively impacted from the washing forces, such as exhibiting a total electrical continuity loss due to complete delamination. This behavior did indeed validate the suitability of conductive threads as a material for integrated electrical interconnects on a textile.

Lastly, we approached the studied resistance-per-meter increase cautiously, as the precision requirements of the planned application could in fact determine whether the observed changes were within the tolerance limits of the end product, and did not negatively impact its expected lifetime or performance, as we observed cases where the advertised R/m almost doubled after tension, such as the HC40, which can be an obstacle to power budget calculations, can lead to resonant frequency changes, and add losses among other things.

## 5. Future Work

It would be interesting to further explore the effects of tension on the performance of conductive threads in real-life tension inducing cases. For example, when they are part of a flexible garment, after a user performs sports activities, etc. Only then can accurate conclusions be drawn on their stability for usage in daily life applications. Additionally, it would be of interest to explore the applicability of such threads in the transmission of digital data and the impact of mechanical stresses on their capability, and lastly see whether their behavior is being impacted from ambient humidity, human sweat, etc.

## 6. Conclusions

Following experimentation with the whole conductive-thread catalog of the two leading thread suppliers, it was found that—in general—the elongation at break tended to be underestimated in the datasheets while the force at break was overestimated. Additionally, the most stable thread of the population tested was the HC 40 by Madeira among the seven conductive-thread series tested. One of the most puzzling results was the “two-stage” breakdown of the Silver-tech 50, and the fact that even after minimal tension, not destructive enough to permanently alter the length of the threads, their resistivity rapidly increased, as a sizable portion of their silver coating was either damaged or completely lost in the process. An interesting variation in the destruction patterns in the case of permanent breakage and permanent deformation was also observed, the former of which potentially led to non-linearity effects on the thread.

In terms of washing, it was found that even though the electrical properties of the threads were only impacted in the form of an expected resistance-value change, the material properties seemed to change quite significantly, with the threads generally becoming stiffer and tolerating less force at break, along with lesser elongation. Of course, this result was valid only on the specific conditions of the washing test and could not be assumed a general finding.

Therefore, the interested parties in working with such threads, usually through automated machine embroidery, should be very careful and characterize their end products, as various tension points on the threads while embroidering (tension knobs, presser feet, needles, etc.) might alter the performance of the chosen thread, and if the application is precision-based, might render the end product defective. It is also advised to include some form of washing performance testing if the application would be directly on a wearable garment.

## Figures and Tables

**Table 1:** Key thread parameters from manufacturer datasheets.

	Manufacturer/Series	Thread	Linear Density (dtex) (DIN EN ISO 2060)	Breaking Force (N) (DIN EN ISO 2062)	Elongation at Break (%) (DIN EN ISO 2062)	Resistance
Polyamide/ Polyester Hybrid	AMANN Silver-tech (S)	30	320 × 3	29	20	<85 Ω/m
	AMANN Silver-tech (S)	50	210 × 3	19.3	20	<150 Ω/m
	AMANN Silver-tech (S)	120	93 × 3	12.6	16	<530 Ω/m
Polyamide	AMANN Silver-tech+ (S+)	100	110 × 3	10.3	35	<200 Ω/m
	AMANN Silver-tech+ (S)	150	110 × 2	7	35	<300 Ω/m
	Madeira HC	12	610 ± 15	29.5	22	<100 Ω/m
	Madeira HC	40	290 ± 6	14	21	<300 Ω/m

**Table 2:** Goodness of fit data per fitted line and thread type.

Thread		Average	Minimum	Maximum
S 30	R^2^	0.9372	0.9354	0.9390
	Sy.x	5.510	5.640	5.324
S 50	R^2^	0.4531	0.4531	0.4557
	Sy.x	23.13	23.16	22.86
S 120	R^2^	0.8885	0.8889	0.8794
	Sy.x	5.513	5.522	5.378
S+ 100	R^2^	0.8372	0.8373	0.8420
	Sy.x	12.61	12.59	12.36
S+ 150	R^2^	0.9214	0.9222	0.9271
	Sy.x	7.997	7.941	7.617
HC 12	R^2^	0.9523	0.9521	0.9549
	Sy.x	4.677	4.702	4.513
HC 40	R^2^	0.9591	0.9591	0.9616
	Sy.x	3.355	3.359	3.230

**Table 3:** Comparison of theoretical versus tested values for maximum force at break and percentage of elongation at break for all tested threads.

	Thread	Theoretical Breaking Force (N) (DIN EN ISO 2062)	Experimental Breaking Force (N) (DIN EN ISO 2062)	Theoretical Elongation at Break (%) (DIN EN ISO 2062)	Experimental Elongation at Break (%) (DIN EN ISO 2062)
Polyamide/ Polyester hybrid	S 30	29	33.6	20	27.92
	S 50 ^1^	19.3	17.6	20	35.6
	S 120	12.6	12.45	16	19.28
Polyamide	S+ 100	10.3	10.2	35	36.64
	S+ 150	7	6.45	35	32.56
	HC 12	29.5	27.8	22	25.36
	HC 40	14	14.1	21	21.08

^1^ For this thread, the force at break was considered as the point of total fracture.

**Table 4:** Tension-exerting force causing permanent damage per thread.

Thread	Average Force (N) (±5% of Max Force)	Percentage of Maximum Force at Break (%)
S 30	13.44 ± 1.68	35
S 50	8.8 ± 0.88	45
S 120	7.47 ± 0.62	60
S+ 100	6.12 ± 0.51	60
S+ 150	4.515 ± 0.32	70
HC 12	11.12 ± 1.39	40
HC 40	6.345 ± 0.705	45

**Table 5:** Resistance measurements per thread, pre- and post-permanent deformation tension.

Ticket No.	Theoretical R (Ω/m)	R before Tension (Ω/m)			R after Tension (Ω/m)		
		Average	Minimum	Maximum	Average	Minimum	Maximum
S 30	<85 Ω/m	102	83	134	202	166	241
S 50	<150 Ω/m	161	154	191	258	201	311
S 120	<530 Ω/m	602	587	633	1031	887	1348
S+ 100	<200 Ω/m	231	212	255	378	299	406
S+ 150	<300 Ω/m	346	317	373	441	402	557
HC 12	<100 Ω/m	132	98	154	276	202	311
HC 40	<300 Ω/m	288	264	331	603	309	771

## Data Availability

Study data can be obtained from the corresponding author upon request.

## Supplementary Materials

The following supporting information can be downloaded at: https://www.mdpi.com/article/10.3390/polym14214581/s1, Figure S1: HC 12 tensioned sample at x400 magnification; Figure S2: HC 12 tensioned sample at x2k magnification; Figure S3: HC 40 tensioned sample at x250 magnification; Figure S4: HC 40 tensioned sample at x2k magnification; Figure S5: S+ 100 tensioned sample at x30 magnification; Figure S6: S+ 100 sample tensioned at x2k magnification; Figure S7: S+ 150 tensioned sample at x30 magnification; Figure S8: S+ 150 tensioned sample at x2k magnification; Figure S9: S 120 tensioned sample at x30 magnification; Figure S10: S 120 tensioned sample at x2k magnification; Figure S11: S 50 tensioned sample at x100 magnification; Figure S12: S 50 tensioned sample at x2k magnification; Figure S13: AFM profile of a healthy sample of S 120; Figure S14: AFM profile of an S 120 sample after 10 wash cycles and tension testing.
