# Supplementary material for: A Study of the Performance Degradation of Conductive Threads Based on the Effects of Tensile Forces and Repeated Washing
Source: Polymers (Basel). 2022 Oct 28;14(21):4581. doi: 10.3390/polym14214581 (PMC9656624; doi:10.3390/polym14214581)
Supplement: Supplementary file 1 [file polymers-14-04581-s001.zip › polymers-1935103-SI.pdf]

Supplementary Material:

Figure S1: HC 12 tensioned sample at x400 magnification
Figure S2: HC12 tensioned sample at x2k magnification
Figure S3: HC 40 tensioned sample at x250 magnification
Figure S4: HC 40 tensioned sample at x1k magnification
Figure S5: S+ 100 tensioned sample at x30 magnification
Figure S6: S+100 sample tensioned at x2k magnification
Figure S7: S+150 tensioned sample at x30 magnification
Figure S8: S+150 tensioned sample at x2k magnification
Figure S9: S 120 tensioned sample at x30 magnification
Figure S10: S 120 tensioned sample at x2k magnification
Figure S11: S 50 tensioned sample at x100 magnification
Figure S12: S 50 tensioned sample at x2k magnification
Figure S13: AFM profile of a healthy sample of S 120
Figure S14: AFM profile of an S120 sample after 10 wash cycles and tension testing

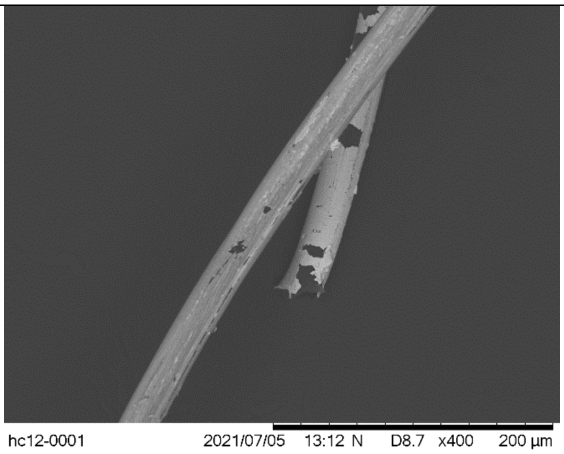

Figure S1: HC 12 tensioned sample at x400 magnification

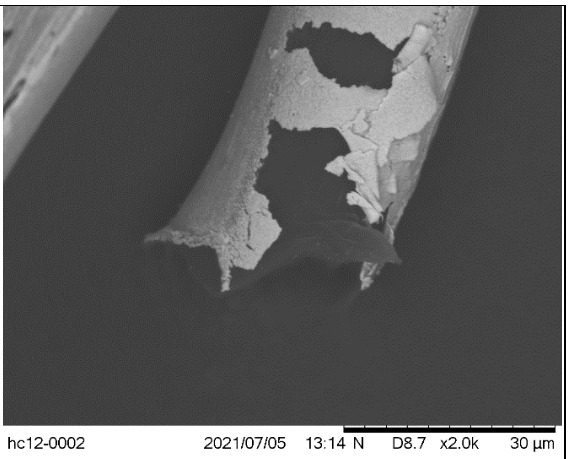

Figure S2: HC12 tensioned sample at x2k magnification

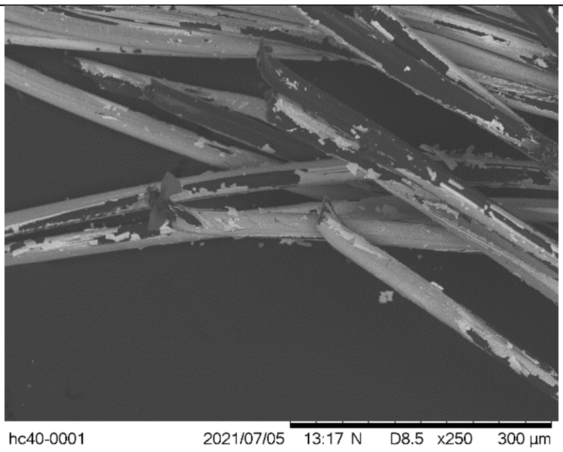

Figure S3: HC 40 tensioned sample at x250 magnification

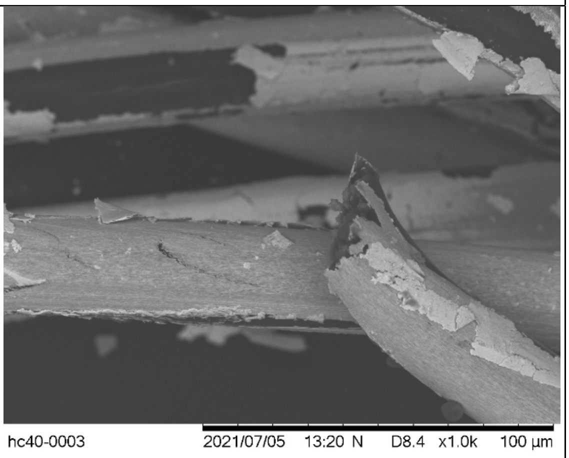

Figure S4: HC 40 tensioned sample at x1k magnification

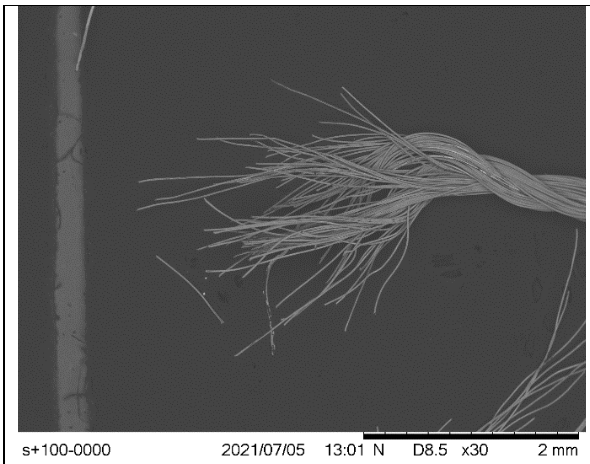

Figure S5: S+ 100 tensioned sample at x30 magnification

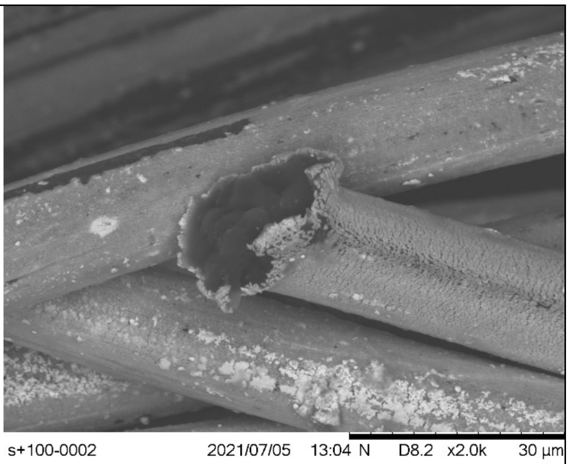

Figure S6: S+100 sample tensioned at x2k magnification

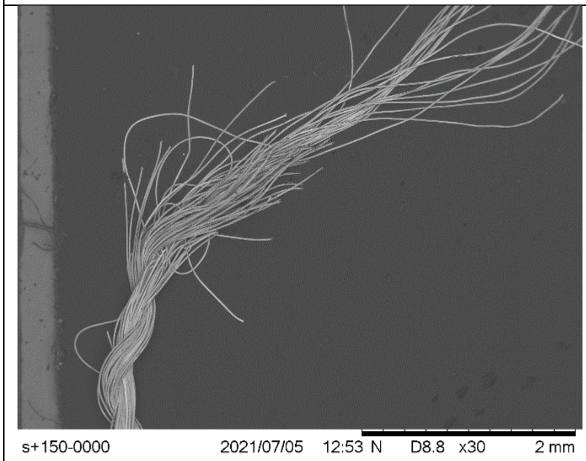

Figure S7: S+150 tensioned sample at x30 magnification

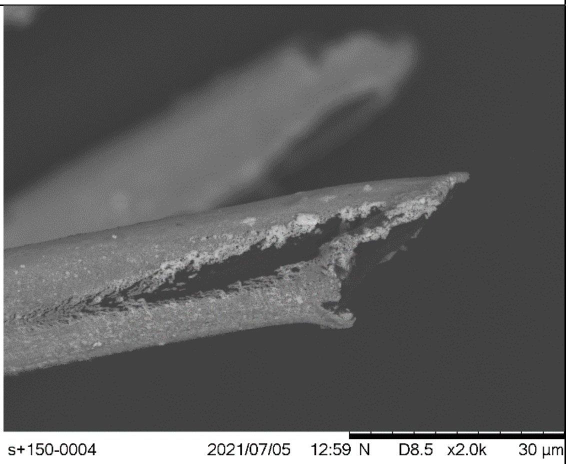

Figure S8: S+150 tensioned sample at x2k magnification

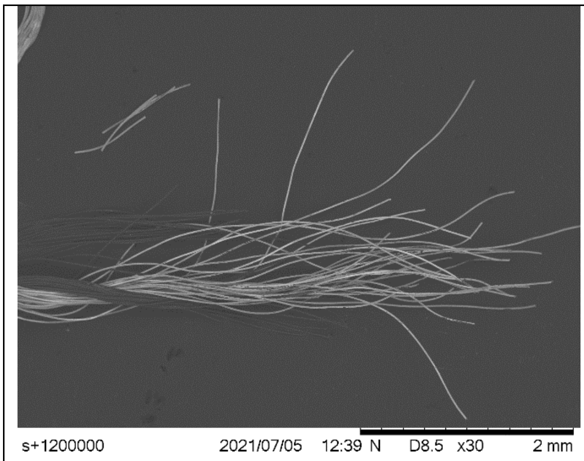

Figure S9: S 120 tensioned sample at x30 magnification

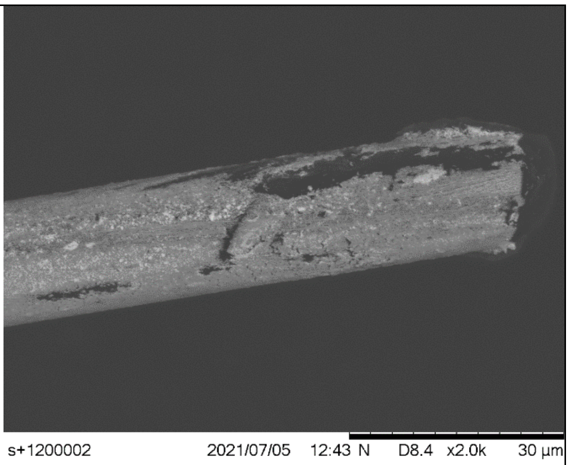

Figure S10: S 120 tensioned sample at x2k magnification

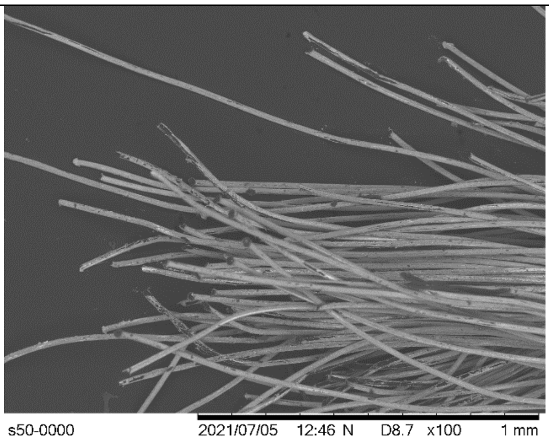

Figure S11: S 50 tensioned sample at x100 magnification

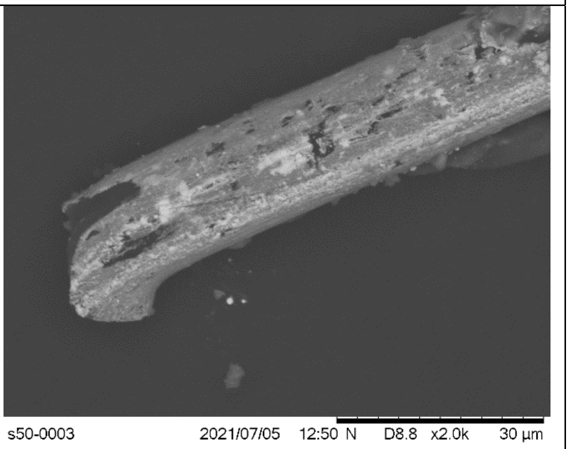

Figure S12: S 50 tensioned sample at x2k magnification

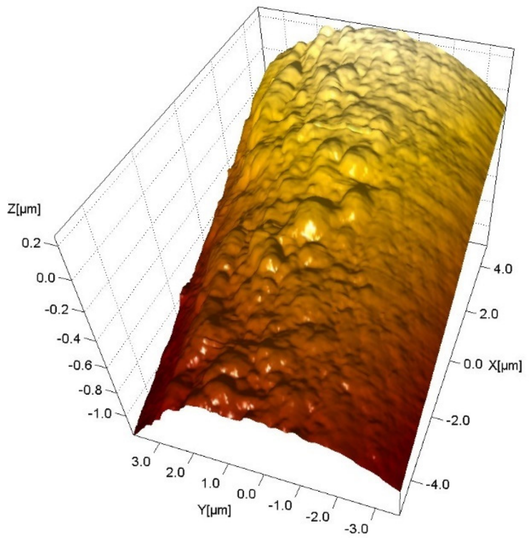

Figure S13: AFM profile of a healthy sample of S 120

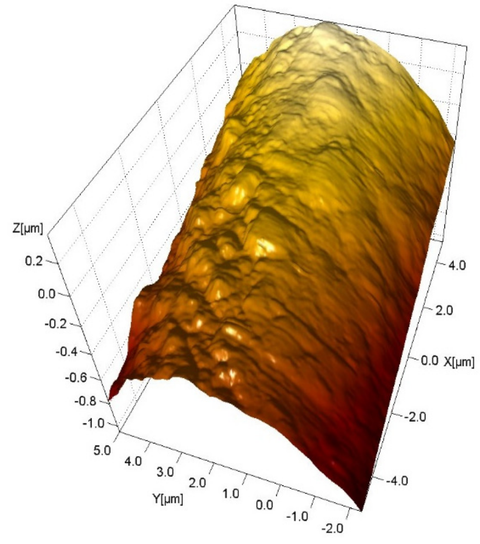

Figure S14: AFM profile of an S120 sample after 10 wash cycles and tension testing
